# Precision Controllable Reversible Watermarking Algorithm for Oblique Photography 3D Models

**DOI:** 10.3390/s26010243

**Published:** 2025-12-30

**Authors:** Ruitao Qu, Liming Zhang, Zhaoyang Hou, Mingwang Zhang

**Affiliations:** 1 Faculty of Geomatics, Lanzhou Jiaotong University, Lanzhou 730070, China; 13240118@stu.lzjtu.edu.cn (R.Q.); 13230085@stu.lzjtu.edu.cn (Z.H.); 13230098@stu.lzjtu.edu.cn (M.Z.); 2 National-Local Joint Engineering Research Center of Technologies and Application for National Geographic State Monitoring, Lanzhou 730070, China; 3 Key Laboratory of Science and Technology in Surveying & Mapping, Lanzhou 730070, China

**Keywords:** precision controlled, reversible watermarking, 3D models, oblique photography, robustness

## Abstract

Most oblique photography 3D model watermarking algorithms only support limited data recovery or fail to restore the original model, falling short of meeting diverse user needs. Consequently, this study introduces a novel reversible watermarking scheme specifically tailored for oblique photographic 3D models, which is designed to adjust the accuracy of model recovery freely. Firstly, considering the global stability of the oblique photography 3D model, the feature points are extracted by utilizing the mean angle between vertex normals. Secondly, a mapping is established based on the ratio of distances between feature points and non-feature points. Then, the vertices are grouped, with each group consisting of one feature point and several non-feature points. Finally, by using the feature point as the origin, a spherical coordinate system is constructed for each group. The watermark information is embedded by modifying the radius in the spherical coordinate system. In the process of extracting watermarks, watermarks can be extracted from different radius ranges, thereby achieving a controllable error in model recovery. Experimental results demonstrate that this approach exhibits significant advantages in reversibility and controllable restoration accuracy, achieving error-free extraction under both translation and rotation attacks. Compared to existing algorithms, it achieves average improvements of 0.121 and 0.298 under cropping and simplification attacks, respectively, showcasing enhanced robustness. This enables it to meet better diverse user demands for watermarking and model restoration in oblique photography 3D models.

## 1. Introduction

In the last two decades, 3D modeling based on oblique photogrammetry has become the development trend of 3D urban construction [1,2,3]. Oblique photogrammetry technology is widely used in fields such as positioning services, building modeling, protecting cultural heritage, and urban landscape planning due to its high efficiency, wide application scope, and Mineral monitoring [4,5,6,7,8]. The oblique photography 3D model is a 3D model obtained by using oblique photography technology to capture surface features. The 3D models can provide high-precision terrain and feature information, which has a high production cost and use value [9]. However, during data applications, it is often accompanied by illegal infringement and risk of leakage. To meet the copyright protection and precision requirements of oblique photography 3D models, it is necessary to research technical means that can balance copyright protection and precision control. This endeavor will foster data sharing and better facilitate the construction of digital cities.

Digital watermarking technology is considered an effective solution for protecting the copyright of 3D models [10,11]. In the research of geospatial data security, digital watermarking technology has been primarily applied to vector geographic data [12,13,14,15] and remote sensing images [16,17,18,19]. However, the research on data protection methods for 3D models is still in its early stages. According to the embedding methods of 3D model digital watermarking algorithms, these methods can be divided into irreversible watermarking algorithms [20,21] and reversible watermarking algorithms [22,23]. There have been a considerable number of research achievements in this area.

For example, Ohbuchi et al. first proposed a digital watermarking algorithm for three-dimensional models [24]. The algorithm utilizes the geometric, topological, and sequential relationships between points, lines, polygons, or surface combinations to embed watermark information. This algorithm validates the feasibility of the embedded digital watermarking algorithm, but the robustness of the algorithm is insufficient. Ren et al. proposed an information hiding algorithm based on 3D model texturing and structural data [25]. They first selected texture maps to hide secrets using a 2D discrete Daubechies transform, then employed STL models as carriers to hide secrets in the wavelet domain, thereby enhancing the algorithm’s robustness. Jiao et al. proposed a method involving point cloud clustering to calculate and extract the centroids and feature points of each cluster. Watermarks are embedded into each cluster, with their positions determined by the distance between feature points and the cluster center. Texture coordinates from oblique photogrammetry 3D models are embedded to enhance the robustness of the models [26]. Nan et al. proposed a multi-level curvature and weighted distance grouping-based oblique-projection 3D model watermarking algorithm [20]. First, PCA calculates curvature to select feature points. Then, these points are grouped based on a weighted composite distance. Finally, height difference normalization is used as the carrier, with the watermark embedded using the QIM method. Zhang et al. proposed a robust watermarking algorithm based on Mahalanobis distance (MD) and feature point extraction, comprising an MD-based zero watermarking algorithm and an ISS feature point watermarking algorithm [21]. First, MD is calculated to construct a feature matrix and generate a zero watermark. Subsequently, ISS feature points are extracted, and their information is embedded into the watermark.

As mentioned above, when designing irreversible watermarking algorithms, it is necessary to control the impact of watermark embedding on the model and emphasize the robustness of the algorithm. However, irreversible algorithms inevitably cause a loss of data accuracy. Therefore, reversible watermarking algorithms with lossless properties have gradually gained attention from researchers. There is limited research on reversible watermarking algorithms for oblique photography 3D models data, but some scholars have made attempts. For instance, Wang et al. combined reversible watermarking algorithms with 3D models [27]. The algorithm embedded watermarks by modulating the distance from a vertex to the centroid of its neighboring vertices while maintaining the topological structure unchanged. This provides insights into reversible watermarking algorithms for oblique photography 3D models. Peng et al. proposed a visible reversible watermarking algorithm for 3D models, which exhibits robustness against translation, rotation, scaling, mesh subdivision, and mesh smoothing [22]. Qiu et al. developed a reversible watermarking algorithm based on quick response (QR) codes [23]. It supports rich-information watermark embedding and demonstrates good robustness against both geometric and non-geometric attacks. However, it does not consider the control of precision during model recovery.

Based on the above analysis, the current research on irreversible watermarking algorithms generally emphasizes robustness but ignores the impact of watermark embedding on data accuracy. On the other hand, reversible watermarking algorithms can recover the original model and overcome this limitation. However, these methods often do not consider the issue of controlling data precision. Therefore, this paper proposes a novel reversible watermarking algorithm for oblique photography 3D models that offers controlled data precision restoration.

The rest of this paper is organized as follows: Section 2 elaborates on the fundamental principles of feature point extraction, vertex grouping, and watermarking image Arnold scrambling. In Section 3, we provide a detailed explanation of the proposed watermarking scheme for oblique photography 3D models. Finally, Section 4 presents the performance study, and Section 5 offers a conclusion of this paper.

## 2. Preliminary

### 2.1. Feature Point Extraction

**Figure 1:**
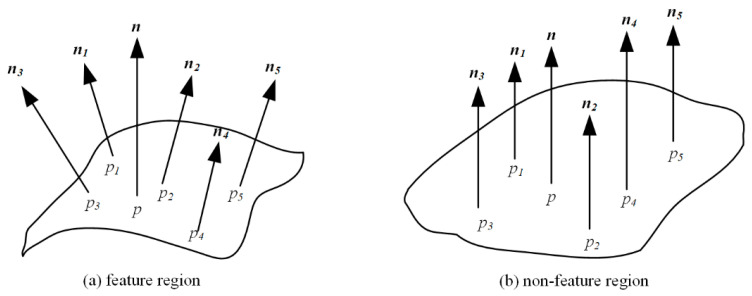
Normal vector diagram.

The feature points of oblique photography 3D models represent the fundamental geometric or texture features of the 3D model. These points are stable global features that remain unchanged under coordinate system transformations. To enhance the algorithm’s robustness and reliability, it employs a feature point extraction method based on the average angle of local surface normals. The normals of the 3D model’s vertices, along with representations of feature and non-feature regions, are illustrated in Figure 1. By extracting all points from the oblique photogrammetric 3D model, a vertex set is obtained. Subsequently, feature points are extracted by calculating the average angle of these point normals.

The primary steps of this method are as follows.

Step 1: Calculate the normal vector $\vec{n_{i}}$ for the points within the vertex set P of the oblique photography 3D model.

Step 2: Calculate the angle $\theta_{pq}$ between the normal vectors of a point and its k-nearest neighbors, taking point p as an example. The expression for this weight is presented in Equation (1).

(1) $$\theta_{pq}=\cos^{-1}\left(\frac{x_{p}x_{q}+y_{p}y_{q}+z_{p}z_{q}}{\sqrt{{x_{p}}^{2}+{y_{p}}^{2}+{z_{p}}^{2}}\sqrt{{x_{q}}^{2}+{y_{q}}^{2}+{z_{q}}^{2}}}\right)$$

where the $\left(x_{p},y_{p},z_{p}\right)$ denotes the coordinates of the normal vector of point p, $\left(x_{q},y_{q},z_{q}\right)$ denotes the coordinates of the normal vectors of the other points in the k-neighborhood.$\;q\in \left\{1,2,\ldots ,k\right\}$.

Step 3: Compute the mean angle $\bar{\theta }$ between the normal vectors of point p and its k-nearest neighbors in Equation (2).

(2) $$\bar{\theta }=\frac{1}{k}\sum_{q=1}^{k}\theta_{pq}$$

Step 4: Set an appropriate threshold ε, when $\bar{\theta }\geq \varepsilon$, it is considered that the point and its neighborhood have a significant variation, which indicates good feature representation. Therefore, these points are classified as feature points. Conversely, when $\bar{\theta }<\varepsilon$ it is considered that the point and its neighborhood have a small variation, indicating a relatively flat surface and poor feature representation. Thus, these points belong to non-feature points.

### 2.2. Vertex Grouping

Based on previous experiments, we have observed that the feature points of oblique photography 3D models remain unchanged before and after embedding a watermark, while the non-feature points undergo minimal modifications. This part ensures the stability of point grouping during the watermark embedding process. To further improve the robustness of the watermark algorithm, the vertices of oblique photography 3D models are divided into multiple groups. Meanwhile, watermark embedding and extraction are performed within each group.

The extracted feature points of oblique photography 3D models are divided into several groups based on the spatial distance ratio between points. $f_{1}=\{p_{i},0\leq i\leq m-1\}$ and $f_{2}=\{p_{j},0\leq j\leq n-1\}$ denote the sets of feature points and non-feature points,, respectively. Here, m and  n represent the number of feature points and non-feature points respectively. A mapping relationship is established between $f_{1}$ and $f_{2}$ based on the distance ratio, and grouping is performed accordingly.

Taking the i-th feature point $p_{i}$ in the set $f_{1}$ as an example, the steps for vertex grouping are as follows:

Step 1: Select the feature point $p_{i}$ as the current object, iterate through each point $p_{j}$ in the non-feature point set. Calculate the spatial distance ratio from $p_{i}$ to $p_{j}$. The mathematical equation of the ratio is as follows:

Step 2: Find several non-feature points with the minimum distance ratios and sort them in ascending order based on their Ratio values. Select the smallest few non-feature points as corresponding points to ensure that each non-feature point corresponds to a unique feature point.

Step 3: Establish a correspondence between the feature point $p_{i}$ and the identified corresponding non-feature point $p_{j}$. Based on this mapping relationship, group the feature points. Each group contains a feature point and its corresponding number of non-feature points.

Step 4: Repeat the steps mentioned above by selecting the next feature point as the current object and continue searching for the corresponding relationship from the remaining non-feature point set. Repeat this process until all feature points have been grouped.

### 2.3. Watermark Image Arnold Scrambling

**Figure 2:**
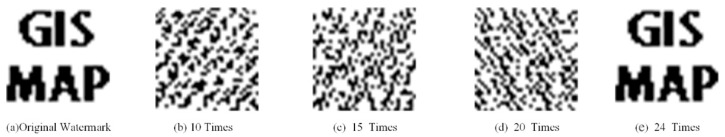
Watermark images.

To enhance the security of the original watermark information, the watermark image undergoes Arnold scrambling transformation. The calculation formula is depicted in Equation (3). In this paper, the original copyright information is a binary image of size 32 × 32 pixels, containing the text “GIS MAP”. In this experiment, the scrambling period for the watermark image is 24 iterations. Meanwhile, the watermark image returns to its original state after the 24th iteration of scrambling, which is shown in Figure 2.

(3) $$\left[\begin{array}{l} x^{\prime }\\ y^{\prime } \end{array}\right]=\left[\begin{array}{l} 1\;1\\ 1\;2 \end{array}\right]\left[\begin{array}{l} x\\ y \end{array}\right]\mathrm{mod}(L),x,y\in \left\{0,1,2,\cdot \cdot \cdot ,L-1\right\}$$

where $\left(x,y\right)$ represents the coordinates of a pixel in the original image, while $\left(x^{\prime },y^{\prime }\right)$ represents the coordinates of the pixel in the image after applying the Arnold transform. “mod” denotes the modulo operator and L represents the length of the watermark.

## 3. The Proposed Algorithm

**Figure 3:**
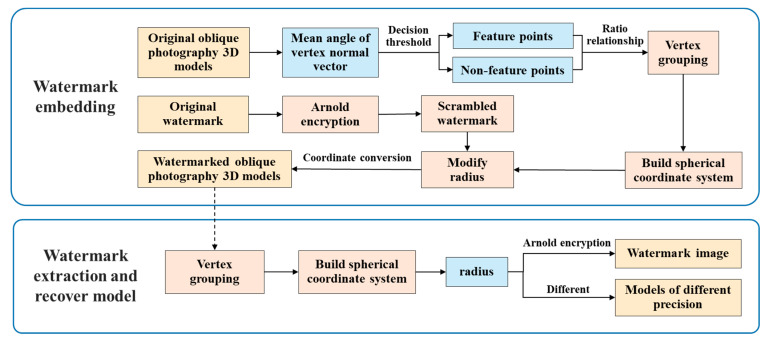
The flowchart of reversible watermarking algorithm.

The algorithm presented in this article is composed of two segments:

(1) Watermark embedding stage. First, the watermark image is preprocessed by Arnold transform. Secondly, feature points are extracted based on the mean angle between the vertex normal vectors. Then the distance ratio between feature points and non-feature points is divided into groups, and the spherical coordinate system of the two types of points is constructed in the groups. Finally, the scrambled watermark information is embedded into the 3D models by modifying the radius of the spherical coordinate system;
(2) Watermark extraction and model recovery stage. The data processing process is consistent with the early stage of embedding. The spherical coordinates are obtained by obtaining the grouping and conversion calculation of feature points, extracting the watermark using the obtained radius value, and then restoring the original model through different radius values obtained from different positions. The detailed flowchart of this algorithm is depicted in Figure 3.

### 3.1. Watermark Embedding Algorithm

The specific steps of the reversible watermarking embedding algorithm based on feature point grouping are as follows:

Step 1: In the grouping, the rectangular coordinates of all non-feature points are converted to spherical coordinates using the corresponding feature points as the coordinate origin. The right-angle coordinates of feature points and non-feature points are denoted as $p_{i}=(x_{i},y_{i},z_{i})$ and $p_{j}=(x_{j},y_{j},z_{j})$, j∈{0≤l≤n−1}, and n denotes the number of non-feature points in the grouping, respectively, and then the conversion formula is shown in Equation (4).

(4) $$\left\{\begin{array}{l} r_{l}=\sqrt{{\left(x_{j}-x_{i}\right)}^{2}+{\left(y_{j}-y_{i}\right)}^{2}+{\left(z_{j}-z_{i}\right)}^{2}}\\ \theta_{l}=\arctan\left(\frac{y_{j}-y_{i}}{x_{j}-x_{i}}\right)\\ \varphi_{l}=\arccos\left(\frac{z_{j}-z_{i}}{r_{l}}\right) \end{array}\right.$$

where $r_{l}$, $\theta_{l}$ and $\varphi_{l}$ denote the distance, azimuth angle, and polar angle between $p_{i}$ and $p_{j}$, respectively.

Step 2: Embed the watermark into the spherical coordinates of the grouped non-feature points, as depicted in Equation (5). Determine the watermark position with the radius $r_{l}$ derived from the non-feature point $p_{j}$ by $w_{i}=w\left[r_{l}{\%w}_{length}\right]$, “[·]” means rounding, and $w_{length}$ means the watermark length. Then $w_{i}$ is embedded into the radius $r_{l}$ of the corresponding non-featured point to reach the new radius ${r^{\prime }}_{l}$, when embedding the watermark.

(5) $${r^{\prime }}_{l}=\frac{Int(r_{l}\times 10^{t})+\left[w_{i}+Modf(r_{l}\times 10^{t})\right]/10}{10^{t}}$$

where *t* represents the embedding position of the watermark information within the radius in spherical coordinates, Int and Modf represent the functions for obtaining the integer and fractional parts, respectively.

Step3: During the embedding process, the transformation of coordinates is applied to the quadratic operation result ${r^{\prime }}_{l}$, to obtain the Cartesian coordinates. The calculation formula is shown in Equation (6), where parameter t determines the embedding position of the watermark, allowing for multiple embeddings.

(6) $$\left\{\begin{array}{l} x^{\prime }=x_{i}+{r^{\prime }}_{l}\times \sin\theta_{l}\times \cos\theta_{l}\\ y^{\prime }=y_{i}+{r^{\prime }}_{l}\times \sin\theta_{l}\times \sin\varphi_{l}\\ z^{\prime }=z_{i}+{r^{\prime }}_{l}\times \cos\varphi_{l} \end{array}\right.$$

where $\left(x^{\prime },y^{\prime },z^{\prime }\right)$ denotes the rectangular coordinates of the non-featured point $p_{j}$ containing the watermark information.

### 3.2. Watermark Extraction Algorithm

Watermark extraction is the reverse process of watermark embedding. For oblique photography 3D models embedded watermark information, the watermark extraction process can be summarized in the following steps:

Step 1: Use the methods described in Section 2.1 and Section 2.2, feature points are extracted based on the mean angle of local vertex normal vectors. The extracted feature points are then grouped with non-feature points to obtain point grouping information.

Step 2: During the point grouping, following the approach discussed in Section 2.2, the Cartesian coordinates of non-feature points are transformed into spherical coordinates. The corresponding $\left({r^{\prime }}_{l},{\theta^{\prime }}_{l},{\varphi^{\prime }}_{l}\right)$ values are calculated accordingly.

Step 3: Watermarks are extracted from each group, where the watermark position index corresponding to ${r^{\prime }}_{l}$ follows the same calculation method used during the embedding process. Here, t represents the location where the watermark was embedded, as given in Equation (7).

(7) $${w^{\prime }}_{i}=\left[Modf({r^{\prime }}_{l}\times 10^{t})\times 10\right]$$

### 3.3. Recover Model Algorithm

The calculations of $\left({r^{\prime }}_{l},{\theta^{\prime }}_{l},{\varphi^{\prime }}_{l}\right)$ between the two types of points are obtained during the watermark extraction stage. Then, we calculate $r_{l}$ by Equation (8). By obtaining different $r_{l}$ values from different t values, we can calculate the corresponding Cartesian coordinates $\left(x,y,z\right)$, thus achieving the restoration of the original model at different levels of precision. The solving formula is given in Equation (9).

(8) $$r_{l}=\frac{Int({r^{\prime }}_{l}\times 10^{t})+\left[Modf({r^{\prime }}_{l}\times 10^{t+1})\right]}{10^{t}}$$

(9) $$\left\{\begin{array}{l} x=x_{i}+r_{l}\times \sin\theta_{l}^{\prime }\times \cos\theta_{l}^{\prime }\\ y=y_{i}+r_{l}\times \sin\theta_{l}^{\prime }\times \sin\varphi_{l}^{\prime }\\ z=z_{i}+r_{l}\times \cos\varphi_{l}^{\prime } \end{array}\right.$$

## 4. Experiment and Analysis

**Figure 4:**
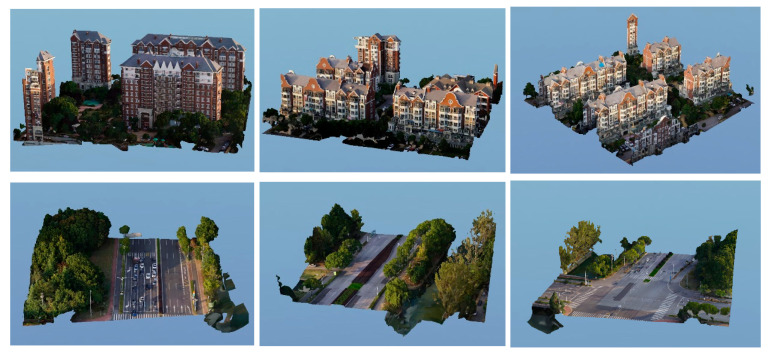
Six of the experimental oblique photography 3D models.

To verify and evaluate the watermarking algorithm proposed in this paper, we selected six oblique photography 3D models shown in Figure 4.

The basic attribute information of each experimental model is depicted in Table 1, including the number of points, the number of triangular meshes, and the range of coordinate values. Additionally, the primary parameter settings in the analog experiment are as follows: k = 35, ε  = $60^{{}^{\circ}}$.

### 4.1. Invisibility Analysis

It is invisible that data changes caused by embedding watermarks in data processing cannot be perceived. After the watermark is embedded in oblique photography 3D models, the degree of change in the model before and after is judged from two aspects: subjective visual analysis and objective indicator analysis.

#### 4.1.1. Subjective Visual Analysis

**Figure 5:**
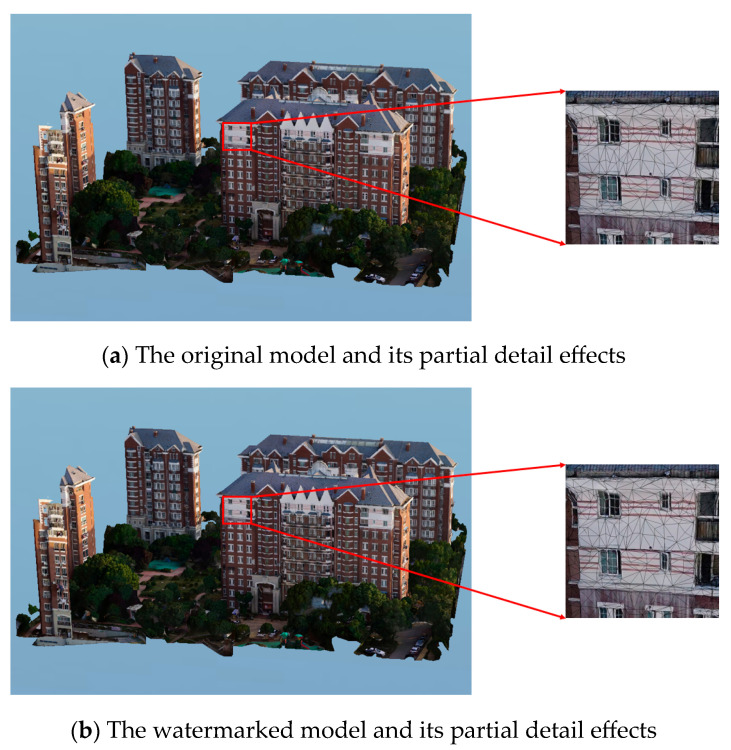
Details of oblique photography 3D models before and after watermark embedding.

Using Model_01 as an example, conduct a subjective visual analysis. Based on the partial detail comparison of the models before and after watermark embedding (Figure 5), it becomes evident that the changes in the triangular meshes are almost imperceptible when zoomed in. The data alterations caused by watermark embedding are difficult to notice within the range of human visual perception, proving that the algorithm exhibits good invisibility.

#### 4.1.2. Objective Indicator Analysis

To further evaluate the invisibility in terms of objective metrics, this paper employs three evaluation metrics: Hausdorff distance (HD), peak signal-to-noise ratio (PSNR), and root mean square error (RMSE). The calculation formulas for these three parameters are Equation (10), Equation (11), and Equation (12), respectively. HD represents the distance between vertex sets of the model before and after embedding the watermark, where a smaller distance indicates a higher similarity between the point sets and greater closeness. PSNR and RMSE are quality assessment metrics borrowed from the field of signal processing, which are used to measure the changes in vertex coordinates of the oblique photogrammetry 3D model before and after watermark embedding. A higher PSNR value implies that the watermark embedding has a lesser impact on data error. RMSE, as an error measurement metric, evaluates the variation difference between two similar oblique photogrammetry 3D models. A smaller RMSE suggests fewer errors and distortions in the model after watermark embedding.

These metrics, HD, PSNRand RMSE, provide quantitative assessments of the watermarking algorithm’s impact on the model and can be used to evaluate the level of invisibility achieved.

Based on Table 2, we can observe that the average HD value after comparing before and after embedding the watermark is greater than 0.00899. Additionally, the average values of RMSE and PSNR are 0.003113 and 94.10342, respectively. These results suggest that the algorithm exhibits good invisibility based on objective metrics evaluation.

(10) $$H(A,B)=\max\left\{\max\left\{h(a_{i},B)\right\},\max\left\{h(b_{j},A)\right\}\right\},(i,j)\in \left\{0,1,2,\cdot \cdot \cdot ,s-1\right\}$$

(11) $$PSNR=\min\left\{10\times \log10(\frac{N\times \max{\left(v_{i}\right)}^{2}}{\sum_{i=0}^{N-1}{\left({v^{\prime }}_{i}-v_{i}\right)}^{2}})\right\},v=\left\{x,y,z,0\leq i\leq N-1\right\}$$

(12) $$RMSE=\sqrt{\frac{1}{N}\sum_{i=0}^{N-1}\left[{\left({x^{\prime }}_{i}-x_{i}\right)}^{2}+{\left({y^{\prime }}_{i}-y_{i}\right)}^{2}+{\left({z^{\prime }}_{i}-z_{i}\right)}^{2}\right]},0\leq i\leq N-1$$

where $h(a_{i},B)$ represents the distance between all a points in the set A and point b in the set B, and $h(b_{j},A)$ is the same. s is the number of points in the point collection, and the maximum value obtained by H(A,B) is the HD distance. N represents the total number of vertices in the 3D model, $v_{i}$ and ${v^{\prime }}_{i}$ respectively represent the original Cartesian coordinate values and the Cartesian coordinate values with a watermark for the i-th point.

### 4.2. Robustness of the Watermarking Scheme

To validate the robustness of the proposed algorithm, this study conducted attack experiments on watermarked oblique-view 3D models using Model_01 as an example. A comparative analysis was performed with related watermarking algorithms. The extracted watermark image and the original watermark image from the watermarked model were evaluated, using the Normalized Correlation (NC) and the Bit Error Rate (BER) as evaluation metrics. In the experiments, the NC value threshold was set to 0.85, indicating that a value greater than the threshold indicates successful watermark extraction. The BER value represents the ratio of erroneous bits extracted from the watermark to the total number of bits in the original watermark. Its value ranges between 0 and 1, with values closer to 0 indicating better robustness of the watermark. The calculation formulas of NC and BER are Equation (13) and Equation (14), respectively.

(13) $$NC=\frac{\sum_{i=1}^{m}\sum_{j=1}^{n}W_{m}(i,j){W^{\prime }}_{m}(i,j)}{\sum_{i=1}^{m}\sum_{j=1}^{n}{W_{m}}^{2}(i,j)}$$

(14) $$BER=\frac{\sum_{i=1}^{m}\sum_{j=1}^{n}W_{m}(i,j)⊕{W^{\prime }}_{m}(i,j)}{L}$$

where $W_{m}(i,j)$ and ${W^{\prime }}_{m}(i,j)$ denote the original watermark image and the detected watermark image, respectively; ⨁ represents XOR operation; L is the size of the watermark image.

#### 4.2.1. Geometrical Attacks

To evaluate the algorithm’s robustness against geometric attacks, we subject the watermarked model to translation and rotation attacks. After applying translation and rotation to the watermarked oblique photography 3D models, the experimental results are shown in Table 3. From this table, it can be found that the algorithm still successfully extracts the valid watermark, with all extracted NC values being 1 and BER values being 0. Therefore, it can be concluded that the algorithm exhibits good robustness against translation and rotation attacks. However, since the algorithm embeds the watermark in the radius distance between vertices, scaling the model alters the distances between points, which makes it impossible to extract the watermark. Hence, the algorithm is not resistant to scaling attacks.

#### 4.2.2. Cropping Attacks

The watermarked 3D model is subjected to different levels of cropping ranging from 15% to 40%. A conclusion can be drawn that the watermark is extracted from each cropped model. The experimental results are shown in Table 4. The algorithm successfully extracts valid watermarks even from the data cropped by 40%, with an NC value of over 0.9 and a BER value of 10.6445%. The result indicates that the proposed algorithm exhibits strong robustness against cropping attacks.

#### 4.2.3. Simplify Attacks

The simplification of a 3D model is a common attack method that can lead to the loss of watermarked vertices, thus impacting the effectiveness of watermark extraction. From the experimental results presented in Table 5, the algorithm demonstrates good robustness against simplification attacks.

#### 4.2.4. Scaling Attacks

The oblique photogrammetry 3D model reversible watermarking algorithm proposed in this paper centers on constructing a spherical coordinate system by grouping feature points and non-feature points. The spherical coordinate radius of non-feature points serves as the core carrier for watermark information. During the watermark embedding phase, the watermark information is concealed by modifying the numerical values of specific precision bits within the radius. Watermark extraction relies on the precise analysis and restoration of these precision bit values.

In scaling attack scenarios, when attackers perform global scaling operations on 3D models, all vertex coordinates undergo proportional stretching or compression according to a uniform scaling factor. This operation directly causes the spatial distance between feature points and their corresponding non-feature points—i.e., the radius in spherical coordinates—to undergo synchronous proportional changes rather than localized minor perturbations. For example, when a model undergoes a 2× scaling attack, the spherical radii of all non-feature points relative to feature points also increase by a factor of 2. The precision bits of the radius modified during the original watermark embedding are then overwritten or distorted by the scaled values. Since watermark extraction algorithms rely on the precision bit distribution pattern of the original radius to decipher the watermark, the distorted, scaled radius values disrupt this pattern. This prevents accurate identification of the encoded watermark information during extraction, ultimately causing watermark extraction failure.

#### 4.2.5. Comparative Analysis of Robustness

To further validate the practicality and robustness of our method, an experiment with the algorithm’s robustness is compared with other watermarking algorithms from related literature [11,23,26,28,29,30]. The NC results of the seven methods are presented in Table 6. According to the given condition, if the NC is less than 0.6, we consider the algorithm unable to extract the watermark.

As depicted in Table 6, the proposed algorithm in this paper alone is compared with six watermarking algorithms, which exhibit good robustness against geometric attacks such as translation and rotation. However, in terms of scale attacks, these three algorithms perform better. Algorithm [28] embeds watermarks by calculating differences based on Z-coordinate sorting, while Algorithm [29] selects vertical feature lines and embeds watermarks into the proportional relationships between segment lengths within each group. Consequently, these methods fail to extract valid watermarks under simplistic attacks. Algorithm [23] embeds visible watermarks by projecting smooth regions from a 3D mesh model onto a 2D plane for cropping and subdivision, making it susceptible to cropping attacks. Algorithms [26,30] classify vertices, using the radius in spherical coordinates as the watermark embedding domain to construct a category-based spherical coordinate system. However, during scaling, the radius changes, thereby preventing the watermark from being fully extracted.

Overall, compared to the watermark embedding algorithms in the six comparative algorithms, the proposed algorithm exhibits good robustness against common attacks, except for scale attacks.

### 4.3. Reversibility Analysis

We can reach the conclusion based on the watermark extraction stage that the embedded watermark can be removed from the radius of the points, which indicates that the proposed watermark algorithm is theoretically reversible. The error statistics between the recovered models and the original models are shown in Table 7. However, upon comparing the error values, we could observe that there is minimal error between the two types of models. The main reason for this result is the rounding reduction in precision during storage and computation of results on a computer. Considering that the experimental data error is set to eight decimal places, which is achievable at the application level in practical scenarios, and the average change in the recovered models is on the order of 10–12 m. From a practical perspective, the experimental results prove that the algorithm exhibits good reversibility.

### 4.4. Precision Control Analysis

The key aspect of the proposed algorithm in this paper lies in its ability to extract the watermark while recovering models with different precisions based on different t values. Through multiple experimental verifications, a conclusion can be drawn that when t values of 2 and 7 are selected, there is a noticeable difference in precision between the recovered models for different t values. The statistical results of the precision comparison between the model restored based on different t values and the original model are shown in Table 8. When t is set to 2, the average change in the recovered models remains at 10^−7^ m, while t = 7, the average change is around 0.001 m. This result indicates that the algorithm effectively controls the average error change in the recovered models, and maintains it within the same order of magnitude. Thus, the proposed algorithm in this article can achieve control over the precision of data recovery, simultaneously ensuring good reversibility and achieving the precision of data recovery within a reasonable range.

## 5. Large 3D Model Scene Discussion

**Figure 6:**
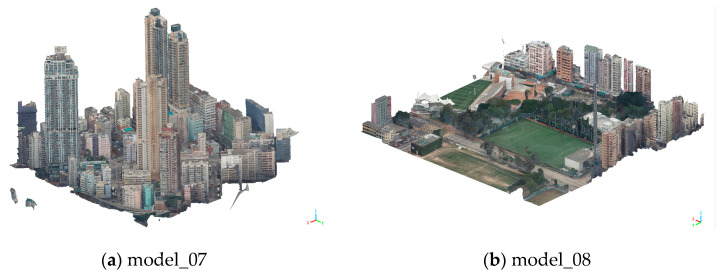
Two experimental large 3D models.

To ensure the experiment’s comprehensiveness and representativeness, two larger-scale 3D models were selected for discussion. All data originated from actual oblique photogrammetry projects, as illustrated in Figure 6. The basic attribute information of the experimental models is presented in Table 9.

### 5.1. Performance Analysis

As the scale of 3D models increases, the resulting rise in geometric complexity and enhanced stability of statistical features leads to an optimized concealment trend for the oblique photogrammetry-based reversible watermarking scheme proposed in this study. Therefore, the subsequent discussion will primarily focus on the robustness and reversibility of the watermarking scheme.

Based on the robustness experiment results in Table 10, both large-scale models demonstrate stability consistent with the small-scale models discussed earlier in terms of resistance to geometric attacks. Against cropping and simplification attacks, the large-scale models demonstrate outstanding robustness: under 15% cropping attacks, Model_07 and Model_08 achieve NC values of 0.996 and 0.995, respectively; under 10% simplification attacks, both models attain NC values of 0.997—significantly exceeding the effective extraction threshold of 0.6. This result demonstrates that the algorithm can accurately locate the watermark embedding region and complete extraction even under partial data loss and data simplification scenarios in large-scale models. Its robustness remains unaffected by model scale expansion, making it suitable for large-scale 3D model applications with high data integrity requirements.

Table 11 demonstrates that the proposed algorithm maintains excellent reversibility even in large-scale 3D models. From a numerical perspective, the recovered models from Model_07 and Model_08 exhibit extremely low errors comparable to those of small-scale models, indicating that increasing model scale does not significantly amplify recovery errors. The primary source of error remains computational rounding precision. The experimental setting of eight decimal places can be reliably achieved in engineering practice, and the recovery errors are far below practical requirements. This demonstrates that the algorithm enables high-precision reversible recovery, satisfying the need for preserving the integrity of large-scale model data.

Table 12 data indicates that the algorithm demonstrates reliable precision control capabilities in large-scale 3D models. At t = 2, the average variation in the recovered models for Model_07 and Model_08 was 3.74832 × 10^−7^ meters and 3.72339 × 10^−7^ meters, respectively. At t = 7, the average variation was approximately 3.7 × 10^−3^ meters, demonstrating the pattern that “lower t values yield higher accuracy,” with all errors remaining controllable. This pattern aligns perfectly with small-scale models, indicating that the algorithm’s precision adjustment mechanism remains unaffected by model scale. By adjusting the t value, it can flexibly adapt to different precision requirements. The consistent error trends across both large-scale models, coupled with their error magnitudes matching those of small-scale models, further validate the algorithm’s precision control and stability.

Based on the comprehensive experimental analysis, the proposed watermarking algorithm demonstrates excellent robustness against common attacks, including translation, rotation, cropping, and simplification in large-scale 3D models, except for scale attacks. Regarding reversibility, the average error between the recovered and original models is on the order of 10^−12^ m, meeting the accuracy requirements for practical applications. Regarding precision control, adjusting the t-value allows flexible control over restoration accuracy, and this adjustment mechanism remains unaffected by model scale. Consequently, the algorithm exhibits excellent scalability, enabling a stable application for watermark embedding and extraction in large-scale 3D models. This provides effective technical support for 3D model copyright protection in fields such as urban 3D modeling and large-scale engineering data security.

### 5.2. Efficiency Analysis

The watermark embedding process comprises four key steps: watermark preprocessing, feature point extraction, vertex grouping, and watermark embedding. The computational complexity of each step directly determines the mapping relationship between efficiency and model scale.

Watermark Preprocessing Stage. Arnold randomization is applied to the watermark image. The computational load is tied to the watermark size and is independent of the 3D model scale, acting as a constant term that does not affect the trend of efficiency as the model grows.

Feature point extraction stage. Distinguishes feature points from non-feature points based on the mean angle between vertex normals. The core computation involves calculating the angle between normals and their mean for points within the k-neighborhood of N vertices, then comparing this to a threshold. The complexity is O(N × k), where k is a constant linearly proportional to the model’s vertex count N.

Vertex grouping stage. Establish unique mappings and group vertices based on distance ratios between feature points and non-feature points, using feature points as centers. Core computation: Iterate through M feature points and N − M non-feature points, compute M × (N − M) distance ratios, and sort them. Since M represents the model’s key points and is significantly smaller than N, feature point density stabilizes. Complexity approximates O(N^2^), making this the primary factor affecting efficiency.

Watermark embedding stage. Construct a spherical coordinate system, modify the radii of non-feature points, and embed the watermark. The core computation involves: performing spherical coordinate transformation, radius modification, and Cartesian coordinate inverse transformation for each non-feature point. The complexity is O(N − M) ≈ O(N), linearly proportional to the model’s vertex count N.

**Figure 7:**
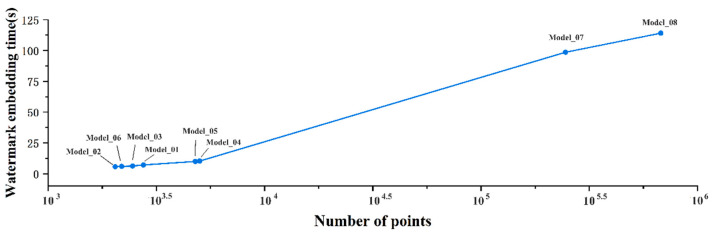
Experimental results of efficiency assessment.

The experimental validation results for the small-scale models Model_01–06 and large-scale models Model_07–08 are shown in Figure 7. When the model scale is small, the number of feature points M increases synchronously with N; however, t the overall cardinality remains low, resulting in a slow growth rate of the O(N^2^) computational complexity for vertex grouping. Linear terms, represented by feature point extraction and watermark embedding, account for a higher proportion of the total computational load, leading to a gradual increase in overall computational complexity. Efficiency is characterized by short embedding times and high performance, with no significant decline trend as model scale increases. As the model scale grows, efficiency should rapidly decrease, primarily driven by vertex grouping complexity. However, since feature point distributions in terrain and buildings follow natural spatial patterns, the density of key feature points in the model remains fixed. Consequently, the growth of feature point count M quickly stabilizes and no longer increases synchronously with N. The actual complexity of vertex grouping degrades from O(N^2^) to O(M × N). Consequently, for large models, watermark embedding time still increases with N, but the rate of increase slows significantly and stabilizes.

Based on the experimental principles, workflow design, and model validation results of the algorithm presented in this paper, the core variation pattern of watermark embedding efficiency with increasing 3D model size is as follows: embedding efficiency exhibits a nonlinear decline trend as model scale increases. However, due to the stabilization of feature point density, the rate of decline significantly slows down and stabilizes once the model scale reaches a certain threshold. Therefore, the proposed method can be effectively applied to large-scale 3D models.

## 6. Conclusions

An innovative digital watermarking algorithm for oblique photography 3D models based on vertex grouping is proposed, integrating precision control with reversible watermarking technology. This algorithm constructs a spherical coordinate system based on the distance ratio relationship between feature points and non-feature points, then calculates the watermark position according to the radius. The watermark is embedded multiple times into the point radii of the 3D model, ensuring stability in vertex distribution and reducing vulnerability to various attacks. Experimental results demonstrate that the proposed algorithm achieves controllable accuracy during model recovery and exhibits robust performance against translation, rotation, cropping, and simplification. Theoretical and experimental validation confirms the algorithm’s reversibility, with the recovered model exhibiting an average error of 10^−12^ m. Compared to existing algorithms, the proposed method offers superior resistance to clipping and simplification attacks, providing effective technical support for 3D model copyright protection in fields like urban 3D modeling. Its only limitation is insufficient resistance to scaling attacks. Future research plans include embedding multiple watermark information across different features or regions and integrating various watermarking techniques to enhance the algorithm’s robustness against scaling and noise attacks.

## Figures and Tables

**Table 1:** Properties of oblique photography 3D models.

3D Model	Number of Points	Number of Meshes	Coordinate Range (x)	Coordinate Range (y)	Coordinate Range (z)
Model_01	2753	2803	−100.15010834, 0.15001816	−0.15010814, 100.15010834	−6.11222792, 30.96369743
Model_02	2060	2039	−0.15034783, 110.7681130	−243.5473909, −143.3426959	−4.61764622, 29.22712517
Model_03	2482	2397	−100.15010834, 0.15001816	−100.15010834, 0.15010814	−4.81751442, 27.58547592
Model_04	5035	7964	−310.65600732, −209.84999084	199.84999084, 281.8805542	−21.88563919, 16.29739952
Model_05	4827	5458	−150.15011597, −49.44667969	−0.15010813, 100.6561439	−19.09381104, 21.78419495
Model_06	2192	2313	−200.5692138, −99.9826490	99.65783235, 200.15011597	−16.38249207, 18.72704315

**Table 2:** Evaluation parameters of invisibility.

3D Models	Evaluation Parameters		
	HD	RMSE	PSNR
Model_01	0.00899423	0.003446	109.02778
Model_02	0.00899958	0.002434	97.57949
Model_03	0.00899835	0.002837	90.85347
Model_04	0.00899777	0.003218	95.85264
Model_05	0.00900007	0.003368	85.73576
Model_06	0.00899944	0.003376	85.57139
mean value	0.00899824	0.003113	94.10342

**Table 3:** Experiment results of geometric attacks.

Attack Type	Attack Intensity	Extracted Watermark	Evaluation Parameters	
			NC	BER
Translation	Translate 60 m along the X-axis	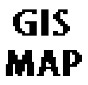	1.000	0.000%
	Translate 60 m along the Y-axis	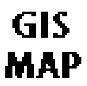	1.000	0.000%
	Translate 60 m along the Z-axis	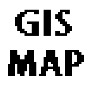	1.000	0.000%
Rotation	Rotate 45° around the X-axis	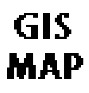	1.000	0.000%
	Rotate 45° around the Y-axis	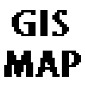	1.000	0.000%
	Rotate 45° around the Z-axis	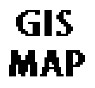	1.000	0.000%

**Table 4:** Experiment results of cropping attacks.

Attack Type	Crop Ratio	Extracted Watermark	Evaluation Parameters	
			NC	BER
Cropping	15%	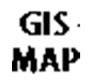	0.996	0.488%
	25%	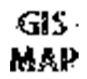	0.965	4.883%
	40%	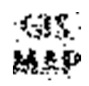	0.926	10.645%

**Table 5:** Experimental results of simplification attacks.

Attack Type	Simplify Ratio	Extracted Watermark	Evaluation Parameters	
			NC	BER
Simplify	10%	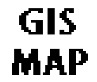	0.996	0.195%
	15%	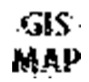	0.972	3.906%
	20%	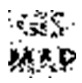	0.907	13.086%

**Table 6:** Comparative analysis of experimental results.

Attack Type and Intensity	Wang [28]	Gong [29]	Qiu [23]	Lee [11]	Jiao [26]	Wang [30]	Our
Translate 60 m along the X-axis	1.000	1.000	1.000	1.000	1.000	1.000	1.000
Rotate 45° around the X-axis	1.000	1.000	1.000	1.000	1.000	1.000	1.000
Scaling 10%	1.000	1.000	1.000	1.000	0.483	0.427	0.444
Crop 15%	0.970	0.987	0.388	0.925	0.981	0.989	0.994
Simplify 10%	0.511	0.493	0.961	0.834	0.674	0.528	0.996

**Table 7:** Error statistics between recovered models and original models.

Recovered Models	t Takes the Values of 2 and 6 Sequentially		
	Maximum Change (Unit: m)	Minimum Change (Unit: m)	Average Change (Unit: m)
Model_01	1.81659 × 10^−11^	3.55271 × 10^−15^	3.69755 × 10^−12^
Model_02	4.79689 × 10^−11^	1.42108 × 10^−14^	1.44995 × 10^−11^
Model_03	1.67708 × 10^−11^	7.10542 × 10^−15^	5.05048 × 10^−12^
Model_04	1.68946 × 10^−11^	1.42108 × 10^−14^	1.42108 × 10^−12^
Model_05	2.51867 × 10^−11^	8.88178 × 10^−16^	6.84313 × 10^−12^
Model_06	1.32921 × 10^−11^	7.10542 × 10^−15^	3.42056 × 10^−12^
Numerical mean	2.30465 × 10^−11^	5.04779 × 10^−15^	5.82205 × 10^−12^

**Table 8:** Precision statistics with different t-values between recovered models and original models.

3D Model	t	Maximum Change (Unit: m)	Minimum Change (Unit: m)	Average Change (Unit: m)
Model_01	2	8.99975 × 10^−7^	2.21011 × 10^−11^	3.88603 × 10^−7^
	7	8.99418 × 10^−3^	1.54276 × 10^−7^	3.88174 × 10^−3^
Model_02	2	8.99973 × 10^−7^	2.43159 × 10^−11^	3.85782 × 10^−7^
	7	8.99940 × 10^−3^	2.69523 × 10^−8^	3.86710 × 10^−3^
Model_03	2	8.99966 × 10^−7^	2.22341 × 10^−11^	3.85321 × 10^−7^
	7	8.99833 × 10^−3^	1.02956 × 10^−6^	3.86065 × 10^−3^
Model_04	2	8.99739 × 10^−7^	3.79203 × 10^−12^	3.87015 × 10^−7^
	7	8.99773 × 10^−3^	2.04559 × 10^−7^	3.88255 × 10^−3^
Model_05	2	8.99671 × 10^−7^	6.46154 × 10^−12^	3.90780 × 10^−7^
	7	8.99998 × 10^−3^	8.62768 × 10^−7^	3.89817 × 10^−3^
Model_06	2	8.99590 × 10^−7^	1.91879 × 10^−11^	3.92975 × 10^−7^
	7	8.99941 × 10^−3^	2.53038 × 10^−7^	3.84723 × 10^−3^

**Table 9:** Properties of large 3D models.

3D Model	Number of Points	Number of Meshes	Coordinate Range (x)	Coordinate Range (y)	Coordinate Range (z)
Model_07	672,505	1,338,287	−949.884, −694.049	−705.810, −449.974	−84.8697, 179.869
Model_08	245,551	485,084	−209.864, 45.9749	−192.188, 63.6513	−85.8668, 118.284

**Table 10:** Experimental Results of Large 3D Model Robustness Analysis.

Attack Type and Intensity	Model_07	Model_08
Translate 60 m along the X-axis	1.000	1.000
Rotate 45° around the X-axis	1.000	1.000
Scaling 10%	0.463	0.452
Crop 15%	0.996	0.995
Simplify 10%	0.997	0.997

**Table 11:** Error statistics between recovered models and original models for large-scale 3D models.

Recovered Models	t Takes the Values of 2 and 6 Sequentially		
	Maximum Change (Unit: m)	Minimum Change (Unit: m)	Average Change (Unit: m)
Model_07	1.46744 × 10^−11^	5.27363 × 10^−15^	1.68283 × 10^−12^
Model_08	1.57318 × 10^−11^	7.83217 × 10^−15^	1.47367 × 10^−12^

**Table 12:** Precision statistics with different t-values between recovered models and original models for large-scale 3D models.

3D Model	t	Maximum Change (Unit: m)	Minimum Change (Unit: m)	Average Change (Unit: m)
Model_07	2	8.99917 × 10^−7^	1.91742 × 10^−11^	3.74832 × 10^−7^
	7	8.99832 × 10^−3^	1.46284 × 10^−7^	3.74208 × 10^−3^
Model_08	2	8.99961 × 10^−7^	2.35673 × 10^−11^	3.72339 × 10^−7^
	7	8.99938 × 10^−3^	2.45806 × 10^−8^	3.73345 × 10^−3^

## Data Availability

The code and data associated with this study are available at: https://github.com/RTQ1306/Reversible-Watermarking-Algorithm-for-Oblique-Photography-3D-Models (accessed on 28 December 2025). All the scripts are run in Python 3.9.
